# Mitochondrial Function, Oxidative Stress, Inflammation and Thrombolytic Treatment in Ischemic Stroke

**DOI:** 10.3390/ijms262110289

**Published:** 2025-10-22

**Authors:** Eleonora Kovacheva, Maria Gevezova, Margarita Koeva, Valentina Mihaylova, Vasilka Kormova, Emanuela Kostadinova, Yulia Kostadinova, Maria Kazakova, Victoria Sarafian

**Affiliations:** 1 Department of Medical Biology, Medical University-Plovdiv, 4000 Plovdiv, Bulgaria; 2 Research Institute, Medical University-Plovdiv, 4000 Plovdiv, Bulgaria; 3 Neurology Ward, University Hospital Pulmed, 4000 Plovdiv, Bulgaria

**Keywords:** ischemic stroke, thrombolysis, inflammation, mitochondrial dysfunction, oxidative stress

## Abstract

Ischemic stroke (IS) is a leading cause of mortality worldwide, responsible for approximately 55% of all neurological disabilities. Proper brain function requires continuous energy supply, primarily generated by mitochondria. Cerebral ischemia impairs the functioning of mitochondrial electron transport chain, reducing adenosine triphosphate production and increasing reactive oxygen species. Inflammation also plays a critical role throughout all stroke phases. YKL-40, a pro-inflammatory glycoprotein, serves as a biomarker of macrophage and microglial activation, while YKL-39 regulates autoimmunity, tissue remodeling, and exhibits monocyte chemotactic and pro-angiogenic activity. This study aims to investigate mitochondrial bioenergetics, oxidative stress, and inflammation in IS patients before and after thrombolysis. Bioenergetic parameters were assessed using Mito Stress Test on an Agilent Seahorse analyzer, and YKL-39, YKL-40 and 4-HNE plasma levels via ELISA. Pre-thrombolytic IS patients demonstrated significantly reduced basal respiration and spare respiratory capacity, along with lower plasma YKL-40 levels. In contrast, they exhibited higher plasma YKL-39 concentrations and increased oxidative stress marker 4-hydroxy-2-nonenal compared to post-thrombolytic patients. These findings highlight novel associations between mitochondrial dysfunction, oxidative stress, and inflammation in IS, and suggest that parameters reflecting these processes may serve as potential biomarkers for evaluating disease severity and predicting outcomes.

## 1. Introduction

Stroke remains one of the leading causes of mortality worldwide and is responsible for approximately 55% of all neurological disabilities [1,2,3]. It typically results from a thrombotic or embolic event that obstructs blood flow to a specific brain region, most often due to underlying conditions such as atherosclerosis, arterial dissection, fibromuscular dysplasia, or inflammatory disorders [4]. The precise timing of symptom onset is critical, as it determines the therapeutic window for the administration of intravenous thrombolytics. Another essential component of clinical evaluation is the identification of the underlying etiology, which plays a pivotal role in elucidating the pathogenetic mechanisms of stroke [5].

Due to its high energy demands, the brain is critically dependent on mitochondrial function to maintain neuronal integrity and activity [6]. Recent research highlights that mitochondrial dysfunction during cerebral ischemia and subsequent reperfusion significantly contributes to the extent of neuronal injury [7,8,9]. Ischemic events disrupt mitochondrial electron transport chain (ETC) activity, impair adenosine triphosphate (ATP) synthesis, and promote the generation of reactive oxygen species (ROS), largely due to reduced oxygen and glucose availability [10,11]. The duration of ischemia is a key determinant as mitochondrial function may be reversible following short period of ischemia, while prolonged episodes often result in irreversible mitochondrial damage. Upon reperfusion, oxygen rapidly re-enters brain tissue. However, this sudden influx exacerbates oxidative stress through excessive ROS production, intensifying cellular injury [12].

This imbalance between pro-oxidant and anti-oxidant systems—termed oxidative stress—is a central factor in the pathogenesis of ischemic stroke (IS), contributing to blood-brain barrier (BBB) disruption, neuronal apoptosis, and tissue degeneration [13,14,15]. At physiological levels, ROS play a regulatory role in maintaining oxygen homeostasis and stimulating erythropoietin production. However, excessive ROS can oxidize important macromolecules, resulting in neuronal death and brain edema [13,14,15,16]. Importantly, mitochondria are themselves the primary source of intracellular ROS under pathological conditions [17,18,19,20]. During sustained oxidative stress, electrons may leak from the ETC and react with molecular oxygen, forming superoxide anions (O_2_^−^), which are key mediators of cellular oxidative injury [21].

Moreover, inflammation is a fundamental contributor to stroke pathophysiology, playing a dual role from the acute phase of vascular occlusion to post-ischemic tissue repair [22]. While it serves to eliminate toxic substances and limit injury, inflammation also amplifies ischemic damage, particularly during the recovery phase. Notably, oxidative stress can further stimulate inflammatory pathways, exacerbating neuronal dysfunction and compromising BBB integrity [23].

Chitinase-3-like protein 1 (CHI3L1), also known as YKL-40, is a 40 kDa glycoprotein and a member of the mammalian chitinase-like protein family. It serves as a biomarker for the differentiation and activation of macrophages and microglia [24]. YKL-40 is expressed by various cell types, including macrophages, chondrocytes, and vascular smooth muscle cells [25,26]. It is believed to reflect the brain’s response to injury and neuroinflammatory processes [27]. Furthermore, the protein has been implicated in the formation of atherosclerotic plaques and the development of IS [27]. Circulating YKL-40 in IS patients is mainly released by brain astrocytes and macrophages in response to local neuroinflammation [28]. Thus, its circulating levels directly reflect neuroinflammation [29].

Another member of the same protein family, chitinase-3-like protein 2 (CHI3L2), also known as YKL-39, is a 39 kDa molecule [30] involved in the regulation of autoimmunity and tissue remodeling [31]. Elevated levels of YKL-39 protein expression have been reported in various degenerative conditions, including osteoarthritis, multiple sclerosis, Alzheimer’s disease, and amyotrophic lateral sclerosis [32,33,34]. The protein has demonstrated monocyte chemotactic activity and pro-angiogenic effects. However, to date, there are no published studies evaluating YKL-39 expression—either independently or in combination with YKL-40—in patients with IS.

4-hydroxy-2-nonenal (4-HNE) is a well-established biomarker of oxidative stress [35,36]. It plays a role in the pathogenesis of several chronic diseases, including cancer [37,38], neurodegenerative disorders [39], and diabetes [40]. Elevated plasma levels of 4-HNE-protein adducts have been observed in patients with Alzheimer’s disease, Parkinson’s disease, Huntington’s disease, and amyotrophic lateral sclerosis [41,42,43]. Despite these findings, data on circulating 4-HNE levels in IS patients remain scarce. It has been suggested that 4-HNE-protein complexes may trigger autoimmune responses and contribute to the progression of neurodegenerative diseases [44,45].

We hypothesize that thrombolytic therapy may contribute to the restoration of mitochondrial function through several interrelated mechanisms. By lysing the occluding thrombus, reperfusion rapidly restores oxygen and nutrient delivery to ischemic neurons, allowing the ETC to resume ATP production and partially reverse ischemia-induced mitochondrial depolarization. Reperfusion may also activate endogenous mitochondrial quality control pathways, including mitophagy and biogenesis, facilitating the removal of damaged mitochondria and the generation of functionally competent organelles. Additionally, the reduction of ischemia-associated ROS production following reperfusion could attenuate oxidative modifications of ETC complexes and membrane lipids, thereby preserving mitochondrial integrity. Finally, reperfusion-induced improvements in bioenergetics may limit secondary activation of inflammatory cascades, reducing further mitochondrial injury and promoting a more favorable cellular environment for neuronal survival. Collectively, these processes may underpin the early post-thrombolytic recovery of mitochondrial respiratory capacity and bioenergetic reserve observed in ischemic stroke patients.

**Figure 1:**
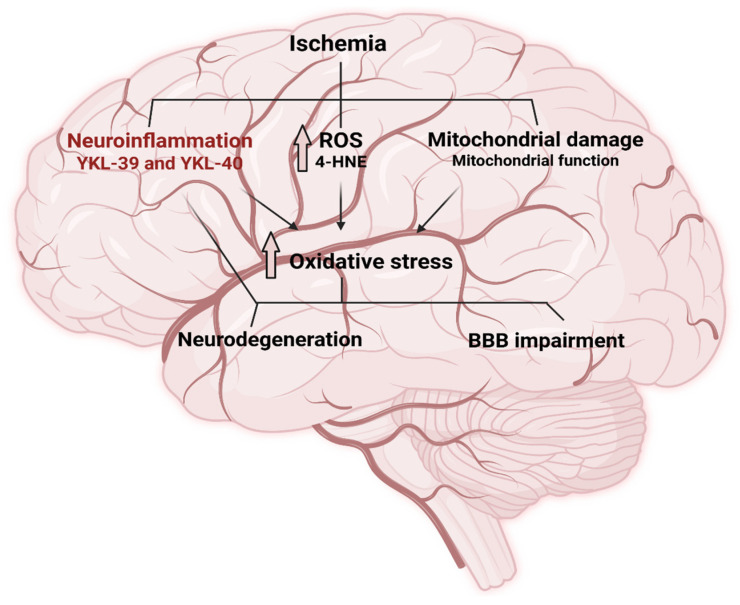
Overview of the complex network between mitochondrial bioenergetics, oxidative stress, and inflammation. The scheme illustrates how ischemic injury disrupts mitochondrial function, leading to impaired ATP production and excessive generation of reactive oxygen species (ROS). ROS in turn trigger oxidative stress pathways, which promote neuroinflammation through activation of microglia, release of pro-inflammatory cytokines, and recruitment of peripheral immune cells. These interconnected processes create a self-amplifying cycle that exacerbates neuronal injury and influences stroke outcomes (Created with BioRender.com, accesses on 4 June 2025). Blood-brain barrier (BBB).

The aim of the present study is to investigate dynamic changes in key parameters of mitochondrial bioenergetics, oxidative stress, and inflammation in IS patients before and after thrombolytic therapy. Our hypothesis is based on the concept that cerebral ischemia induces mitochondrial dysfunction, leading to excessive ROS production, which may cause oxidative damage either directly or through lipid peroxidation, generating molecules such as 4-HNE. High oxidative stress levels result in BBB disruption, neuronal degeneration, and neuroinflammation. Moreover, ischemic events may alter the expression of YKL-39 and YKL-40, which are closely related to inflammatory processes in the central nervous system (CNS) (Figure 1).

## 2. Results

### 2.1. Patients

Sixteen IS patients, who received thrombolytic treatment according to the recommendations, were followed for 24 h. Within the studied patient cohort there is a male predominance (75%) compared to females (25%). All IS patients have arterial hypertension, underscoring its central role as a major modifiable risk factor. Hyperlipidemia was also highly prevalent (93.75%), followed by diabetes mellitus (62.5%) and obesity (37.5%). A smaller proportion had a history of prior stroke (18.75%). At admission, the mean National Institute of Health Stroke Scale (NIHSS) score was 7.69, indicating predominantly moderate stroke severity, while the mean Glasgow-Liege Coma Scale (GLCS) score of 19.5 suggests that most patients maintained preserved consciousness. The biochemical profile demonstrated elevated mean glucose levels (7.85 mmol/L), which may indicate stress hyperglycemia or poorly controlled diabetes, as well as increased mean total cholesterol (6.03 mmol/L) and triglycerides (1.75 mmol/L), consistent with the high prevalence of dyslipidemia (Table 1).

### 2.2. Mitochondrial Parameters

**Figure 2:**
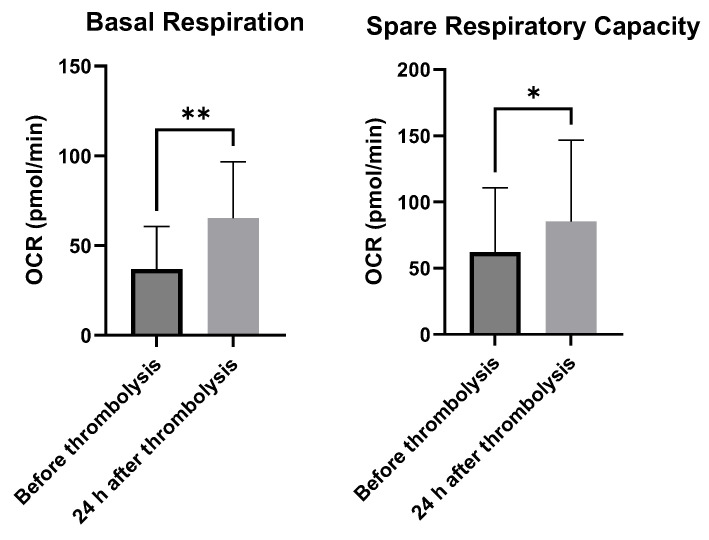
Bioenergetic parameters of ischemic stroke patients. Respiratory metrics of peripheral blood mononuclear cells (PBMCs) by Seahorse assay (Mito Stress Test). Bar graphs showing the differences in bioenergetics parameters between patients before and after thrombolysis, Mann–Whitney—* *p* < 0.05, ** *p* < 0.01.

The metabolic analysis showed that post-thrombolytic IS patients have significantly increased (*p* = 0.0035) basal mitochondrial respiration (65.34 ± 31.40) compared to pre-thrombolytic patients (36.96 ± 23.58). In addition, the SRC is significantly lower (*p* = 0.0317) in patients before thrombolysis (62.15 ± 48.64) and higher in the same patients after treatment (85.31 ± 61.30) (Figure 2).

**Figure 3:**
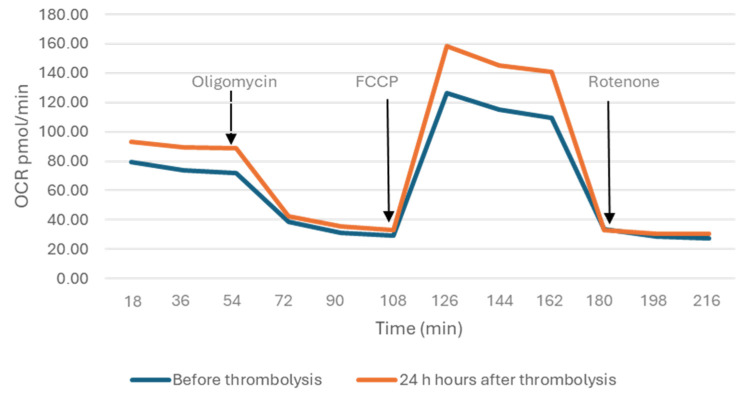
Effect of the used inhibitors (Oligomycin, FCCP, and Rotenone) on the oxidative function of isolated PBMCs.

Figure 3 shows mitochondrial OCR dynamically over time and illustrates the response to sequential inhibitor injections. Twelve measurements over an 18 min period were performed in total.

### 2.3. Plasma Levels of YKL-39, YKL-40 and 4-HNE

**Figure 4:**
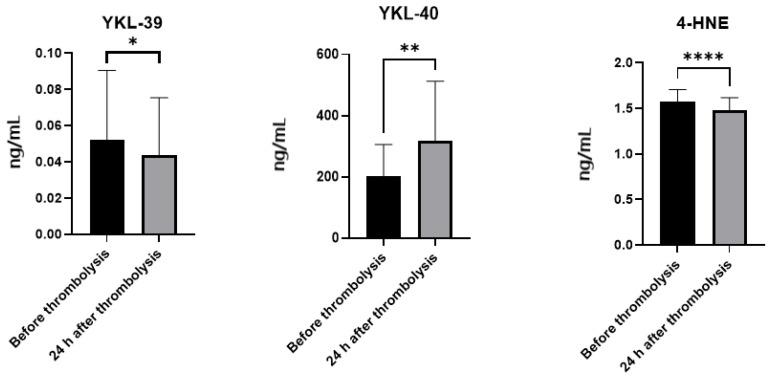
Plasma levels of YKL-39, YKL-40 and 4-HNE in ischemic stroke patients. Bar graphs show differences in plasma concentrations between patients before and after thrombolysis, Mann–Whitney—* *p* < 0.05, ** *p* < 0.01, **** *p* = 0.0001.

IS patients before thrombolysis have significantly higher YKL-39 protein levels (0.052 ± 0.038) compared to post-thrombolytic patients (0.044 ± 0.032) (*p* = 0.0434). The same pattern is observed for plasma levels of 4-HNE, as pre-thrombolytic patients have higher levels (1.575 ± 0.13) in comparison to post-thrombolytic ones (1.481 ± 0.14) (*p* = 0.0001). The levels of YKL-40, on the other hand, increase after therapy (from 200.80 ± 104.70 to 318.60 ± 194.20) (*p* = 0.002) (Figure 4).

Notably, to our knowledge, this is the first study to evaluate circulating YKL-39 levels in the context of IS. The observed reduction following thrombolysis suggests a potential role for YKL-39 as an early inflammatory biomarker that may reflect acute cerebrovascular stress and immune cell recruitment.

The correlation analysis of clinical parameters, mitochondrial respiratory values and plasma levels of YKL-39, YKL-40 and 4-HNE revealed both positive and negative correlations, although the differences were not significant.

## 3. Discussion

### 3.1. Mitochondrial Spare Respiratory Capacity, Basal Respiration and Energetic Adaptation in Ischemic Stroke

Mitochondrial function is inherently dynamic, allowing cells to adapt to fluctuating energy demands through the mobilization of their SRC, which reflects the ability of mitochondria to respond to acute cellular stress or increased energy requirements beyond basal respiration, thereby preventing ATP depletion and preserving cellular homeostasis [46]. As such, it serves as an indicator of mitochondrial resilience and metabolic flexibility [47]. Moreover, SRC is closely associated with mitochondrial plasticity, representing the capacity of the organelles to modulate their bioenergetics in response to pathophysiological challenges [48,49].

Suboptimal SRC levels are commonly linked to impaired mitochondrial function, which may remain undetectable under resting conditions but become evident when energy demand approaches the maximal respiratory threshold [50]. Additionally, mitochondrial quality control mechanisms, including mitophagy and biogenesis, play a critical role in regulating oxidative stress levels, which in turn can negatively affect SRC [51]. Enhanced mitochondrial biogenesis has been shown to increase SRC, supporting the concept that mitochondrial quantity and quality jointly determine cellular energy adaptability [52].

Our findings align with these observations as the SRC levels were significantly reduced in pre-thrombolytic samples compared to post-thrombolytic ones. This suggests that, during the acute ischemic phase, mitochondria are subjected to severe stress, limiting their functional reserve and adaptive capacity. By contrast, 24 h after thrombolytic therapy, SRC was restored to a higher level, potentially reflecting a compensatory response and mitochondrial recovery. The observed post-thrombolytic increase in SRC may therefore represent an adaptive mechanism contributing to cellular resilience in the post-ischemic environment [53].

In addition, basal respiration—defined as the mitochondrial oxygen consumption rate under unstimulated conditions—reflects both ATP-linked respiration and proton leak. In our study, basal respiration was lower in pre-thrombolytic samples compared to post-thrombolytic ones, which may be related to enhanced mitochondrial oxidative phosphorylation following reperfusion. The increased energy demands and improved post-therapy mitochondrial functionality is likely to contribute to the elevation in basal respiration. This observation supports the hypothesis that post-ischemic bioenergetic adaptations serve as a compensatory response to oxidative stress and tissue hypoxia [54].

### 3.2. Neuroinflammation and the Role of YKL-Proteins in Ischemic Stroke

Neuroinflammation represents a pivotal pathophysiological mechanism in ischemic brain injury. Following cerebral ischemia, activation of resident microglia occurs rapidly, accompanied by infiltration of peripheral immune cells into the brain parenchyma within a timeframe ranging from several hours to a few days post-stroke [55,56]. Neutrophils are the first to invade the infarcted region, typically within hours of the ischemic event, followed by monocytes within the initial 24 h, and subsequently by lymphocytes within 24–48 h [57,58]. Monocytes, once recruited, secrete a variety of pro-inflammatory mediators, including chemokines, intercellular adhesion molecules, interleukins (IL-1, IL-6, IL-8), and tumor necrosis factor-alpha (TNF-α), all of which contribute to the amplification of the inflammatory cascade in the ischemic and hypoxic brain environment. Moreover, monocytes facilitate thrombo-inflammatory processes through the formation of platelet-monocyte aggregates, thereby exacerbating vascular occlusion and ischemic damage [59].

Recent findings suggest that YKL-39, a chitinase-like protein, may function as a potent monocyte chemoattractant and angiogenic modulator [60]. Although its role has not been previously characterized in the context of IS, our data indicate elevated plasma levels of YKL-39 in the pre-thrombolytic phase, implying a potential involvement in cerebrovascular remodeling and response to occlusion.

Emerging evidence supports a mechanistic link between bioenergetic regulation and inflammation, as exemplified by the relationship between mitochondrial function and circulating levels of YKL-40 [61]. Upon immune activation, immune cells undergo metabolic reprogramming—transitioning from a quiescent catabolic state to an active anabolic phenotype—a shift that ensures the balance between ATP production and utilization [62,63,64]. YKL-40 has been proposed as a biomarker of neuroinflammation and tissue injury in the CNS [27]. Its expression is modulated by key inflammatory cytokines such as IL-1β and TNF-α, produced by activated macrophages in the context of neuroinflammation [65]. In turn, YKL-40 has been implicated in promoting atherosclerotic plaque formation and may thereby contribute to stroke pathogenesis [66].

In patients with IS, YKL-40 is primarily secreted by astrocytes and macrophages in response to localized neuroinflammation, and its plasma concentrations correlate with the extent of the inflammatory response [28]. Clinical studies have demonstrated that elevated circulating YKL-40 levels are associated with greater stroke severity, as reflected by higher NIHSS scores upon hospital admission [67]. Additionally, YKL-40 exhibits cytoprotective properties, particularly in cardiomyocytes exposed to ischemia-reperfusion injury, by inhibiting apoptosis through suppression of Fas expression via protein kinase B phosphorylation [68].

In our study, YKL-40 concentrations were significantly elevated in post-thrombolytic plasma samples compared to pre-thrombolytic ones, corroborating its role in the post-ischemic inflammatory and repair processes. Notably, the distinct cellular sources of YKL-39 and YKL-40 may underlie their differential expression patterns observed before and after thrombolysis.

Furthermore, during the subacute phase of stroke (hours to days post-event), the sustained infiltration of leukocytes results in the release of pro-inflammatory cytokines, chemokines, and ROS, intensifying the inflammation. This cascade contributes to secondary injury through mechanisms including BBB disruption, cerebral edema, neuronal death, and increased risk of hemorrhagic transformation [69,70].

### 3.3. 4-HNE as a Marker and Mediator of Oxidative Stress in Ischemic Stroke

4-HNE, a highly reactive α,β-unsaturated aldehyde generated during lipid peroxidation, is widely recognized as a reliable biomarker for oxidative stress [71,72]. Among lipid-derived reactive species, 4-HNE exhibits pronounced biological reactivity, contributing significantly to cellular damage under oxidative conditions. It has been demonstrated to impair mitochondrial function by inhibiting adenosine triphosphatase activity and promoting apoptotic signaling, thus disrupting energy homeostasis [71]. In the context of atherosclerosis, 4-HNE accumulates within vascular plaques, where it exacerbates inflammation through the upregulation of pro-inflammatory cytokines such as IL-8 and IL-1β, and may contribute to plaque destabilization and rupture [73].

Elevated levels of 4-HNE have also been reported in myocardial ischemia–reperfusion injury, where its accumulation correlates with the extent of tissue damage [74]. At low concentrations, 4-HNE may exert adaptive roles by initiating antioxidant defense responses. At higher levels, however, it has cytotoxic effects by covalently modifying membrane proteins and essential metabolic enzymes, thereby impairing cellular function [75].

Although 4-HNE targets a variety of cellular components, mitochondria appear to be particularly vulnerable to its effects [71,76]. The ETC, a major source of endogenous ROS, is also one of the primary targets of 4-HNE-mediated damage [71,77,78]. Under physiological conditions, mitochondria possess robust mechanisms to regulate ROS production and maintain redox homeostasis [77]. However, during oxidative stress, these protective systems become overwhelmed, resulting in mitochondrial dysfunction and enhanced susceptibility to lipid peroxidation [77].

4-HNE-mediated mitochondrial injury has been implicated in the pathogenesis of multiple disorders, including Parkinson’s disease, cardiomyopathy, and acute lung injury [71,76,78]. Moreover, the reciprocal relationship between oxidative stress and inflammation further complicates the pathological landscape. When oxidative stress is the initiating factor, it can drive inflammatory responses. Conversely, primary inflammation can potentiate oxidative damage, creating a self-perpetuating cycle that exacerbates tissue injury [79].

Our findings suggest that oxidative stress may represent a primary event following IS, as evidenced by elevated 4-HNE levels in pre-thrombolytic samples. This observation underscores the role of 4-HNE not only as a biomarker, but also as a potential mediator of mitochondrial and inflammatory dysfunction during the acute phase of ischemic insult.

## 4. Materials and Methods

### 4.1. Patients

The study included 16 patients with clinical evidence of IS who were examined on admission to the Neurology ward, before thrombolysis, and 24 h after thrombolysis. During the clinical examination, the severity of neurological symptoms was assessed by a certified neurologist using the NIHSS. The level of consciousness was measured via the GLCS. Vital signs and heart rate were monitored, as well as neck bruising. NIHSS is a standard stroke severity assessment tool that includes 11 categories and scores ranging from 0 to 42. These categories comprise the level of consciousness, gaze direction, vision, facial symmetry, hand and foot motor skills, limb coordination, sensory perception, language ability, clarity of speech and attention to both sides of the body. The scoring was performed in a specific order, with each score based on the patient’s performance at the time of examination rather than predicted ability. A door-to-needle time of 60 min was considered for patients with acute IS who have met the criteria for thrombolysis. GLCS measures three aspects of behavior independently: motor response, verbal presentation, and eye opening. The score of this scale ranges from 3 to 20. Patient’s blood samples were obtained on admission to the Neurology ward and 24 h after administration of the thrombolytic agent. Blood samples collected on admission (prior to thrombolytic therapy) are used to characterize baseline biomarker levels in the acute phase of IS, and the 24 h post-thrombolysis blood samples—to capture treatment-related changes and early subacute biological responses. These timepoints were chosen to reflect clinically relevant phases of stroke evolution.

Inclusion criteria for IS patients comprised a diagnosis of IS and treatment via thrombolysis. The exclusion criteria for IS patients included infection, brain tumor, blood transfusion, atrial fibrillation, and use of contraceptives. Patients with diabetes and hyperlipidemia were included in the study, provided their conditions were stable and controlled at normal ranges under specific treatment. All patients underwent CT-scan examinations at admission to the hospital and 24 h after thrombolysis. All participants enrolled in the survey met the inclusion and exclusion criteria for both clinical assessment scales and signed a written informed consent. The study was approved by the Ethics Committee at the Medical University of Plovdiv (Protocol No 2/07.02.2024).

### 4.2. Isolation of Plasma and Peripheral Blood Mononuclear Cells

Blood samples were collected in EDTA-Vacutainer monovettes in compliance with all conditions for venepuncture. After centrifugation at 800× *g* for 10 min, plasma was aliquoted and stored at −80 °C for further analyses. Peripheral blood mononuclear cells (PBMCs) were isolated using a standard Histopaque gradient solution (Sigma-Aldrich, St. Louis, MO, USA, d = 1.077 g/mL) and then centrifuged according to the manufacturer’s protocol (30 min at 1800 rpm with minimal acceleration and deceleration). The PBMCs’ layer was aspirated into a new 15 mL Falcon tube, mixed with phosphate-buffered saline (PBS), pH 7.4, and centrifuged at 1800× *g* rpm for 10 min. The isolated cells were cultured in RPMI-1640 medium (Pan Biotech Cat NoP04-22100) supplemented with 10% FBS, 1% penicillin/streptomycin in a cell culture incubator overnight at 37 °C, 5% CO_2_ and high humidity. After that, the cells were washed with Seahorse RPMI (Seahorse XF RPMI medium, Agilent Technologies, Inc., Santa Clara, CA, USA, pH 7.4, 500 mL Cat. No 103576-100). Their number and viability were determined using a “LUNA” automated counter (Logos Biosystems, Anyang, Republic of Korea). The cells were plated in poly-D-lysine coated Seahorse assay microplates (Seahorse XFp FluxPak Cat. No 103022-100) at a density of 2 × 10^5^/well and the metabolic analysis was performed.

### 4.3. Analysis of Bioenergetic Parameters of Mitochondria

Oxygen consumption rate (OCR) was measured using the Mito Stress Test Kit on an Agilent Seahorse XFp Analyser (Agilent Technologies, Inc., Santa Clara, CA, USA) according to the manufacturer’s instructions. We used three ETC modulators-oligomycin (1.5 µM), carbonyl cyanide-4-(trifluoromethoxy)-phenylhydrazone (FCCP) (2 µM) and rotenone (0.5 µM). They influence different components of the ETC and mitochondrial respiration, providing information on basal respiration, SRC, coupling efficiency, ATP production, proton leak, and maximal respiration. For each of these parameters, three repeated rates of oxygen consumption measurements were performed over an 18 min period. Firstly, the basal oxygen consumption is measured. Next oligomycin, an inhibitor of complex V, is added, and the resulting OCR is used to calculate ATP-linked respiration and proton leak respiration. After that, FCCP, a protonophore, is added to collapse the inner membrane gradient, driving the ETC to function to its maximum, which allows the calculation of the maximal respiratory capacity. Lastly, rotenone, a complex I inhibitor, is added to shut down ETC function, revealing the non-mitochondrial respiration. The mitochondrial SRC is calculated by subtracting basal respiration from maximal respiratory capacity.

### 4.4. Detection of YKL-39, YKL-40 and 4-HNE Plasma Levels by ELISA

Plasma levels of YKL-39, YKL-40 and 4-HNE were measured using specific commercial ELISA kits—YKL-39 (Circulex, Tessenderlo-Ham. Belgium, Cat. No CY-8087), YKL-40 (Elabscience, Wuhan, China, Cat. No E-EL-H0037) and 4-HNE (Assay Genie, Dublin, Ireland, Cat code: HUFI01747). The measurements were performed in duplicates following the manufacturers’ recommendations. The assay employs a sandwich-based ELISA method with optical density measured at 450 nm on Tecan Sunrise ELISA reader (Tecan Austria GmbH, Salzburg, Austria).

### 4.5. Statistical Analyses

Statistical analyses were performed using GraphPad Prism 10. Descriptive statistics were applied to determine group means and data parametricity. Data were assessed for normality by a Shapiro–Wilk test. Differences between normally distributed variables were evaluated for significance by Welch’s *t*-test or paired *t*-test. For non-normal data, the non-parametric Mann–Whitney assessment was carried out. Values of *p* < 0.05 were considered significant. Results are presented as means ± SD.

## 5. Conclusions

This study provides novel insights into the dynamic interplay between mitochondrial bioenergetics, oxidative stress, and inflammation in IS patients before and after thrombolytic therapy. Our findings demonstrate that ischemia is initially characterized by impaired mitochondrial respiration and elevated YKL-39 and 4-HNE plasma levels, reflecting early neuroinflammation and oxidative stress. Importantly, thrombolysis was associated with partial restoration of mitochondrial respiratory function, as indicated by increased basal respiration and spare respiratory capacity, alongside a reduction in 4-HNE levels. These results suggest that timely reperfusion not only restores cerebral blood flow but may also support mitochondrial recovery and attenuate oxidative injury.

Moreover, the distinct expression patterns of YKL-39 and YKL-40 underscore their potential as complementary biomarkers of neuroinflammation at different stages of stroke evolution. While YKL-39 appears to reflect early monocyte recruitment and cerebrovascular stress, the rise in YKL-40 levels following thrombolysis may represent ongoing astrocytic and macrophage activation linked to tissue repair and remodeling processes. To our knowledge, this is the first report documenting dysregulated YKL-39 and 4-HNE levels in IS, suggesting that these markers could provide additional value for monitoring inflammatory and oxidative responses in the acute and subacute phases.

Taken together, our data support the understanding that oxidative stress represents a primary pathological event in IS, followed by progressive neuroinflammation that evolves during reperfusion and recovery. These findings emphasize the importance of a multidimensional therapeutic strategy targeting not only vascular recanalization but also mitochondrial preservation, oxidative stress reduction, and modulation of the inflammatory response.

## 6. Limitations

Several limitations of this study should be acknowledged. First, the sample size was relatively small (n = 16), which may limit the generalizability of our findings and reduce statistical power to detect subtle associations. Second, the study design included only two timepoints—on admission and 24 h post-thrombolysis—without longitudinal follow-up beyond the first day. As a result, longer-term dynamics of mitochondrial function, oxidative stress, and inflammatory biomarkers could not be assessed, and the temporal relationship with delayed recovery or secondary injury remains unclear. Third, while we measured bioenergetic parameters and plasma biomarkers, the study did not include functional clinical correlations linking these molecular changes to neurological outcomes or stroke severity over time. Therefore, the direct impact of mitochondrial and biomarker alterations on patient prognosis or functional recovery could not be determined. Future studies with larger cohorts, extended follow-up, and integration of clinical outcome measures are warranted to address these gaps and strengthen the translational relevance of our findings.

## Figures and Tables

**Table 1:** Baseline demographic and clinical characteristics of patients with ischemic stroke.

Characteristic	Patients (n = 16)
**Demographic**	
Age (mean ± SD)	70 ± 9.16
Male sex, n (%)	12 (75%)
Female sex, n (%)	4 (25%)
**Risk factors**	
Hypertension (AH), n (%)	16 (100%)
Diabetes mellitus, n (%)	10 (62.5%)
Hyperlipidemia, n (%)	15 (93.75%)
Obesity, n (%)	6 (37.5%)
Prior stroke, n (%)	3 (18.75%)
**Clinical presentation**	
NIHSS score at admission (mean ± SD)	7.69 ± 3.50
GLCS score at admission (mean ± SD)	19.50 ± 1.55
Glucose, mmol/L (mean ± SD)	7.85 ± 3.19
Total cholesterol, mmol/L (mean ± SD)	6.03 ± 1.23
Triglycerides, mmol/L (mean ± SD)	1.75 ± 0.7

## Data Availability

The raw data supporting the conclusions of this article will be made available by the authors on request.

## Abbreviations

The following abbreviations are used in this manuscript:

IS	Ischemic stroke
ETC	Electron transport chain
ATP	Adenosine triphosphate
ROS	Reactive oxygen species
CHI3L2 (YKL-39)	Chitinase-3-like protein 2
CHI3L1 (YKL-40)	Chitinase-3-like protein 1
4-HNE	4-hydroxy-2-nonenal
SRC	Spare respiratory capacity
BBB	Blood-brain barrier
CNS	Central nervous system
NIHSS	National Institutes of Health Stroke Scale
GLCS	Glasgow-Liege Coma Scale
PBMCs	Peripheral blood mononuclear cells
PBS	Phosphate-buffered saline
OCR	Oxygen consumption rate
FCCP	Carbonyl cyanide-4-(trifluoromethoxy)-phenylhydrazone
ELISA	Enzyme-linked immunosorbent assay
SD	Standard deviation
TNF-α	Tumor necrosis factor-alpha
CT-scan	Computed tomography scan
AH	Arterial hypertension
FBS	Fetal bovine serum
